# Combination of Styrylbenzoazole Compound and Hydroxypropyl Methylcellulose Enhances Therapeutic Effect in Prion-Infected Mice

**DOI:** 10.1007/s12035-023-03852-4

**Published:** 2023-12-20

**Authors:** Kenta Teruya, Ayumi Oguma, Sara Iwabuchi, Keiko Nishizawa, Katsumi Doh-ura

**Affiliations:** 1 Department of Neurochemistry, Tohoku University Graduate School of Medicine, 2-1 Seiryo-Machi, Aoba-Ku, Sendai, Miyagi 980-8575 Japan; 2 Faculty of Medical Science & Welfare, Tohoku Bunka Gakuen University, Sendai, Miyagi Japan

**Keywords:** Drug-resistance, Hydroxypropyl methylcellulose, Prion, Styrylbenzoazole, Sustained release

## Abstract

Prion diseases are fatal transmissible neurodegenerative disorders. Tremendous efforts have been made for prion diseases; however, no effective treatment is available. Several anti-prion compounds have a preference for which prion strains or prion-infected animal models to target. Styrylbenzoazole compound called cpd-B is effective in RML prion-infected mice but less so in 263K prion-infected mice, whereas hydroxypropyl methylcellulose is effective in 263K prion-infected mice but less so in RML prion-infected mice. In the present study, we developed a combination therapy of cpd-B and hydroxypropyl methylcellulose expecting synergistic effects in both RML prion-infected mice and 263K prion-infected mice. A single subcutaneous administration of this combination had substantially a synergistic effect in RML prion-infected mice but had no additive effect in 263K prion-infected mice. These results showed that the effect of cpd-B was enhanced by hydroxypropyl methylcellulose. The complementary nature of the two compounds in efficacy against prion strains, chemical properties, pharmacokinetics, and physical properties appears to have contributed to the effective combination therapy. Our results pave the way for the strategy of new anti-prion agents.

**Supplementary Information:**

The online version contains supplementary material available at 10.1007/s12035-023-03852-4.

## Introduction

Prion diseases are fatal neurodegenerative disorders associated with the accumulation of prions in the brain and lymphoid system [1]. They include Creutzfeldt-Jakob disease, Gerstmann-Sträussler-Scheinker syndrome, and fatal familial insomnia in humans. Prions are composed of abnormal prion proteins, which are structural isoforms of normal prion protein; prion propagation involves a conformational change from the normal to the abnormal form at the molecular level, with the abnormal form acting as a seed for the propagation. Various compounds and biological agents have been found to inhibit prion propagation or facilitate prion degradation [2–4].

**Fig. 1:**
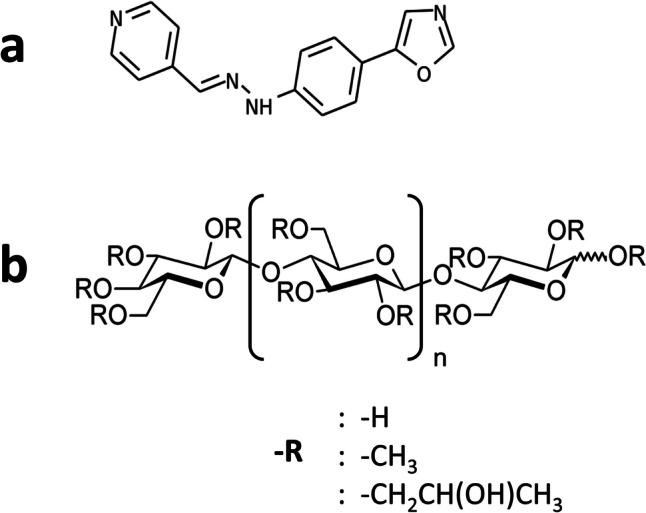
Anti-prion compounds used in the present study.** a** cpd-B. **b** HPMC

Transgenic (Tg) mice overexpressing PrP are frequently used to evaluate the efficacy of anti-prion drugs [5, 6]. Prion-infected Tg7 mice [7] and Tga20 mice [8] are representative and feasible disease models for observing drug-induced incubation period prolongation [5]. Our earlier anti-prion dosage regimen required intracerebroventricular administration for adequate efficacy [9], and we had been searching for compounds that can be administered by less invasive dosing. We found two promising compounds that significantly prolonged the incubation period of prion-infected mice; small organic amyloidophilic compound called cpd-B (1-(4-(oxazole-5-yl)phenyl)-2-((pyridine-4-yl)methylene)hydrazine) [10] (Fig. 1a) and water-soluble polymer hydroxypropyl methylcellulose (HPMC) [11, 12] (Fig. 1b).

Cpd-B is the first compound identified to prolong the incubation period of prion-infected mice when administered orally [10]. Treatment by ad libitum feeding cpd-B-containing food exerts a significant effect against RML prion-infected Tga20 mice but a very limited effect against 263K prion-infected Tg7 mice. Conversely, a single subcutaneous administration of HPMC has excellent efficacy against 263K prion-infected Tg7 mice, but very limited efficacy against RML prion-infected Tga20 mice [12–15]. Thus cpd-B and HPMC exhibited distinctive impacts on RML-Tga20 and 263K-Tg7 mice, respectively. The shorter incubation periods of these prion-infected Tg mice are crucial for determining the sustained anti-prion effects of HPMC. For these reasons, these pairs of Tg mice were employed to examine the combined action of cpd-B and HPMC. Incidentally, cpd-B and HPMC are complementary not only in their molecular properties but also in effectiveness and dosing regimen. Based on these complementary characteristics, this study aimed to investigate the effects of the combination of cpd-B and HPMC in 263K prion-infected Tg7 mice and RML prion-infected Tga20 mice, expecting a synergistic anti-prion effect.

## Materials and Methods

### Animals

Tg7 mice and Tga20 mice were kindly provided by Dr. Bruce Chesebro of the NIAID’s Rocky Mountain Laboratories (Hamilton, MT, USA) and Dr. Charles Weissmann of the Scripps Research Institute (La Jolla, CA, USA), respectively. Tg7 mice were derived from a hamster-prion-protein overexpressing mouse line crossed with a prion-protein-null mouse line and have a mixed genetic background of C57BL/10 and 129/Ola [8]. Tga20 mice are a mouse-prion-protein overexpressing mouse line derived from a prion-protein-null mouse line in a C57BL/6 J background [7].

### Compounds

Cpd-B was synthesized at the Tokyo R&D Center of Daiichi Pharmaceutical Co., Ltd. (Tokyo, Japan) and kindly provided by the company. TC-5RW were used as representative HPMCs of the type E (hypromellose 2910) as classified by the United States Pharmacopeia and kindly provided by Shin-Etsu Chemical Co., Ltd. (Tokyo, Japan). To prepare TC-5RW films containing cpd-B in the animal study, cpd-B (10 mg) was dispersed in 1.0 mL of TC-5RW in 50% (v/v) ethanol–water solution (50 mg/mL) and dried in a plastic weigh dish (4 × 4 cm^2^) for 24 h under atmospheric conditions. TC-5RW films without cpd-B were prepared similarly. Before subcutaneous administration of films, they were completely dried in desiccators under reduced pressure at 25 °C for 24 h.

### Animal Studies

Animal experiments were performed as described previously [14]. Six- to 10-week-old male mice were used in all experiments. The mice were randomly allocated to each experimental group. Prior to any treatments or inoculations, a combination anesthetic containing 0.3 mg/kg of medetomidine hydrochloride (Nippon Zenyaku Kogyo Co., Ltd., Fukushima, Japan), 4.0 mg/kg of midazolam (Sandoz, Tokyo, Japan), and 5.0 mg/kg of butorphanol tartrate (Meiji Seika Pharma Co., Ltd., Tokyo, Japan) was administrated by intraperitoneal injection [16]. TC-5RW film alone, TC-5RW film with cpd-B, or cpd-B alone was subcutaneously administered to mice (for bioassay timelines: Fig. S1(A)). Subcutaneous administration was performed through a small incision in the skin on the back, which was sutured after administration. In the control mouse group, a sham operation was performed. One day after administration, intracerebral infection was performed by inoculation with 20 µL of 1% (wt/vol) brain homogenate, prepared from a terminally ill hamster infected with the 263K prion for Tg7 mice or from a terminally ill mouse infected with the RML prion for Tga20 mice. Mice were observed daily until reaching an obvious symptomatic stage of prion disease, where they were either akinetic with a lack of grooming behavior, coordination, and parachute reaction or exhibited a rigid tail, arched back, and 10% weight loss per week. Considering this stage as a humane endpoint, the mice were killed by cervical dislocation, and their brains were collected and fixed in formalin. The primary endpoint of this study was the survival period, defined as the duration from prion infection to the symptomatic humane endpoint stage. Inclusion and exclusion criteria were pre-determined; all mice were included in the statistical analysis except for those that died within a week after inoculation or treatment due to procedural complications.

### Dye Releasing Assay

TC-5RW films (20 mg) containing water-soluble dye methylene blue (4.0 mg) were prepared as described above. Before the dye-releasing assay, all films were completely dried in desiccators under reduced pressure at 25 °C for 24 h. Films containing dye were placed in the insert chamber of Transwell® (6 wells, Corning) with 2 mL of PBS. The diameter of the insert chamber is 24 mm and the bottom polycarbonate membrane has a pore size of 0.4 μm. Then, 3 mL of PBS was added to the wells. The aliquots of the PBS in the well containing dye passed from the chamber were collected at predetermined intervals. Every time samples were collected from the wells, the wells were washed and 3 mL of PBS was freshly added. Samples were collected over 120 h. The experiments were repeated in triplicate. The dye concentrations were evaluated by spectrometric analysis at 660 nm using a Spectra max 190 UV–vis analyzer (Molecular Devices, Sunnyvale, CA, USA). The cumulative absorbances of released dye for each sample were approximated by the following formula: $F\left(t\right)=A(1-{e}^{-kt})$, where *t* is time in the unit of hours, and *A* and *k* are the curve’s maximum value and primary reaction rate in the unit of hour^−1^, respectively.

### Statistical Analysis

Survival rates were calculated using the Kaplan–Meier method, and the difference in incubation periods between the control and respective treatment groups was evaluated using the log-rank test, hazard ratio, and % increase in the mean incubation periods described previously [15]. Statistical significance was set at *p* < 0.05. Data were analyzed for the log-rank test and Cox’s proportional hazards model in R statistical programming language [17] using the “cox.zph” function from the R package “survival” [18]. The proportional hazards assumption was evaluated as shown in Fig. S1.

## Results

### Combination Treatment with cpd-B and HPMC in Prion-Infected Mice

**Fig. 2:**
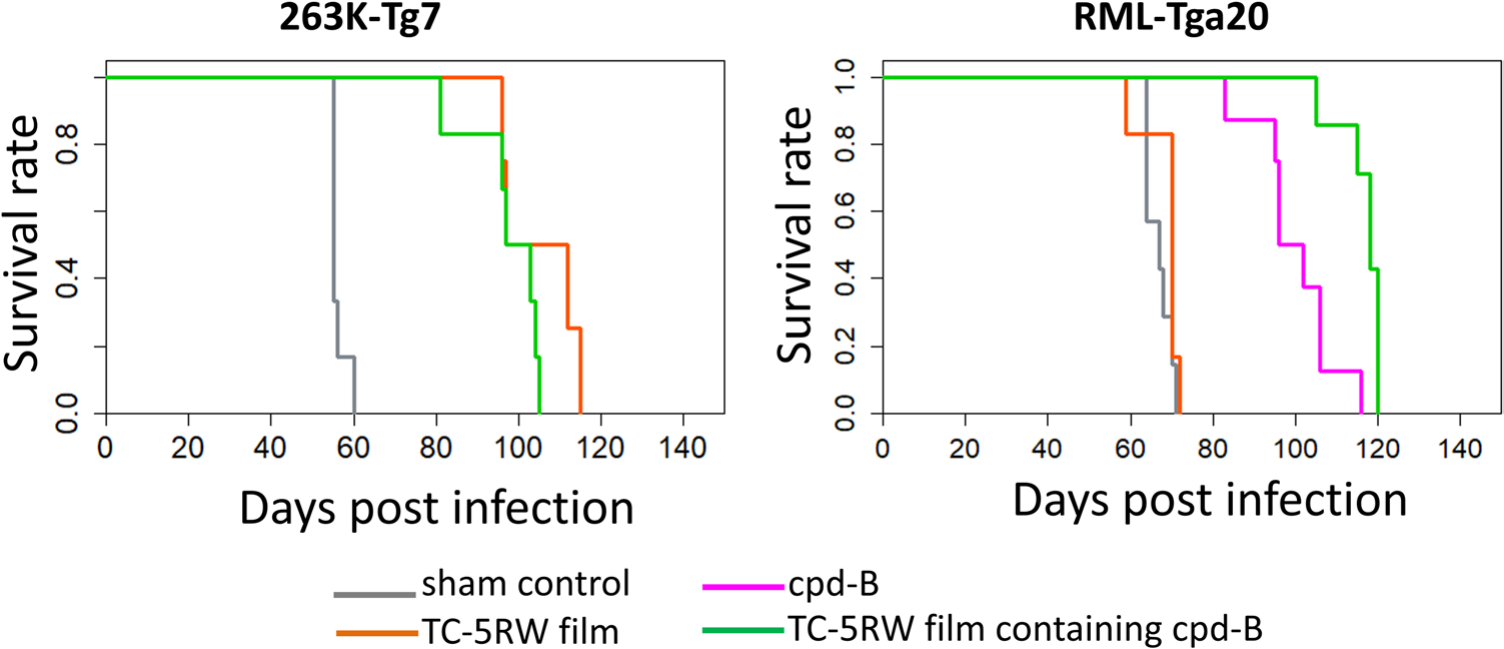
Survival graphs of prion-infected mice treated with the combination of cpd-B and TC-5RW. Kaplan–Meier graphs of 263K prion-infected Tg7 mice and RML prion-infected Tga20 mice are shown

Neither additive nor synergistic effects of the combination of cpd-B and TC-5RW were observed in the 263K prion-infected Tg7 mice. On the other hand, a single subcutaneous. administration of the combination of cpd-B and TC-5RW demonstrated a remarkable extension of the incubation period in the RML prion-infected Tga20 mice (Fig. 2, S1). Compared to the sham control group, the % increase in the mean incubation period of the group of TC-5RW film containing cpd-B was 75%, which was much greater than the sum of those of TC-5RW film alone (2.4%) and cpd-B alone (49%). Accordingly, the combination of cpd-B and TC-5RW exerted a synergistic effect in RML prion-infected Tga20 mice, but not in 263K prion-infected Tg7 mice (Table 1).

**Table 1:** Survival data of prion-infected mice treated with cpd-B and HPMC

Prion strain and mouse	N	Administration^a^	Incubation period (days)		% Increase in mean^b^	Versus sham control		Versus monotherapy^c^	
			Mean ± SD	Median		Logrank *p*	Cox HR	Logrank *p*	Cox HR
263K-Tg7	6	Sham control	56 ± 2.0	55		-	-		
263K-Tg7	4	TC-5RW film	105 ± 9.9	104	88	0.004	3.6 × 10^−10^	-	
263K-Tg7	6	TC-5RW film containing cpd-B	97.7 ± 9.0	100	75	0.0005	2.0 × 10^−10^	0.2	2.6
RML-Tga20	7	Sham control	66.9 ± 3.0	67		-	-		
RML-Tga20	6	TC-5RW film	68.5 ± 4.7	70	2.4	0.3	0.52	-	-
RML-Tga20	8	cpd-B	100 ± 9.8	99	49	5.0 × 10^−5^	4.1 × 10^−10^	-	-
RML-Tga20	7	TC-5RW film containing cpd-B	117 ± 5.4	118	75	1.0 × 10^−4^	5.1 × 10^−10^	0.002	0.11

*N* number of the mouse, *SD* standard deviation, *HR* hazard ratio

^**a**^TC-5RW film alone (50 mg), TC-5RW film (50 mg) containing cpd-B (10 mg), or cpd-B alone (10 mg) was subcutaneously administrated in the mice one day before intracerebral prion-infection.

^**b**^Difference in incubation periods between the sham control and respective treatment groups was evaluated in each set.

^**c**^Calculated against the group of TC-5RW film alone in 263K prion-infected Tg7 mice or the group of cpd-B alone in RML prion-infected Tga20 mice.

Survival curves corresponding to these analyses are shown in Fig. S1(B).

### HPMC Film as Sustained Releasing Material

The ability of HPMC film as sustained-releasing material was characterized using diffusion experiments with water-soluble dye methylene blue. The diffused dye concentration curves for the control solution and TC-5RW film were fitted with first-order reaction rate equations and gave respective rate constants: 1.00 ± 0.036 and 0.511 ± 0.011 h^−1^, with standard errors. Thus, these results suggest that solutes embedded in HPMC films are released slowly depending on the viscosity of HPMC compounds.

## Discussion

Several in vivo pathways capable to modulate the pathogenesis of prion-infected mice have been identified, and combination therapies for prion diseases have been proposed to improve efficacy through synergy [19] [20]. There are a few reports of successful combination therapies in prion-infected animals. Kocisko et al. [21] treated intracerebrally 263K prion-infected Tg7 mice with a combination of pentosan polysulfate [9] and Fe(III) *meso* tetra(4-sulfonatophenyl)porphine [22] and found synergistic effects. Spilman et al. [23] found a synergistic suppression of the atrophy and loss of neuronal dendrites by supplying ad libitum chocolate drinks containing a γ-secretase inhibitor [24] and quinacrine [25] to RML prion-infected CD1 mice.

On the other hand, Abdulrahman et al. [26] observed no additive effects of the combination of subcutaneous administration of HPMC and oral administration of AR12 (OSU-03012, PDK1 inhibitor) [27] to RML prion-infected FVB mice. Burke et al. [28] demonstrated no additive effect but the emergence of drug-resistant prion variant in RML prion-infected mice treated with the combination of IND24 [4] and Anle138b [29], both of which are known to be effective in RML prion-infected mice when administered alone. Therefore, the significance of administering anti-prion compounds in combination is controversial.

In the present study, two compounds with complementary properties were employed on the basis of our previous studies. Cpd-B and HPMC are very different in efficacy against prion strains, chemical properties, pharmacokinetics, and physical properties. Cpd-B has a remarkable potency in administering orally in RML prion-infected Tga20 mice, in which the anti-prion effects of HPMCs are relatively limited even when administered intracerebroventricularly. Cpd-B is an aromatic organic low-molecular-weight compound that is rapidly taken up to the bloodstream and efficiently transferred into the brain but has rapid clearance from the brain [10].

On the other hand, HPMCs retain the strong intermolecular interactions [30] and show high viscosity in aqueous solution. These HPMC properties favor to be processed into films, which are useful as a sustained releasing material [31]. Peripherally administrated HPMCs are distributed to the whole body but much less in the brain, and they retain in the body for a long period. This long retention leads to an excellent prophylactic anti-prion effect [12], which may be a favorable aspect for the prophylaxis of genetic prion diseases [32]. A single peripheral administration of HPMC gives a remarkable extension of the lifespan in 263K prion-infected Tg7 mice, while the anti-prion effect of cpd-B is very limited in the same disease model. The in vivo anti-prion effects of these compounds have been verified also by other research groups [26, 33–35]. Therefore, we expected that even a single subcutaneous administration of HPMC film containing cpd-B would enhance the anti-prion effects in both 263K prion-infected Tg7 mice and RML prion-infected Tga20 mice.

**Fig. 3:**
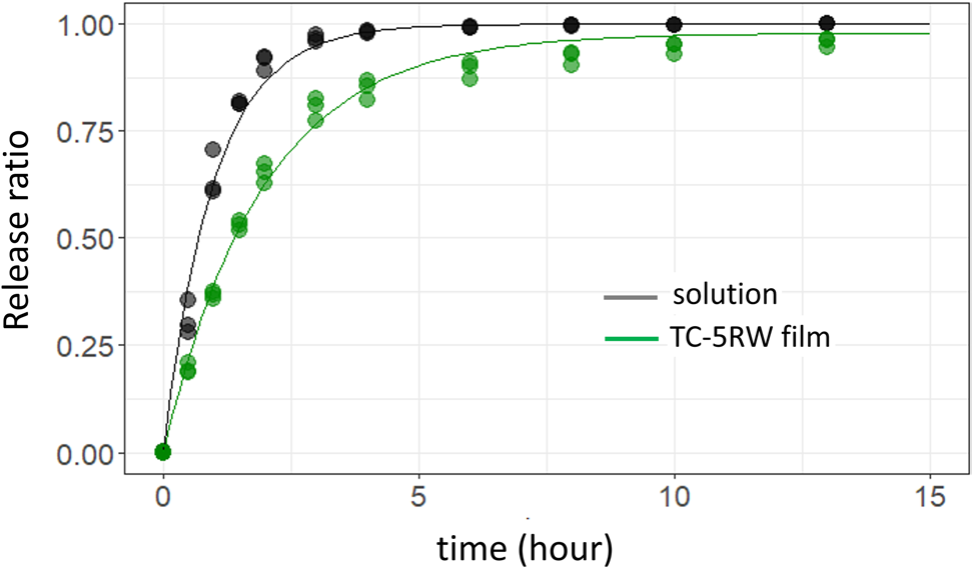
Releasing kinetics of methylene blue from HPMC films. The releasing ratios were calculated from the cumulative absorbance at 600 nm

Indeed, the anti-prion effect of cpd-B in RLM prion-infected Tga20 mice was enhanced by the combination with HPMC film (Fig. 2). Although in vivo molecular mechanism of each compound is still unclear, there are two possibilities for the successful synergistic anti-prion effect in RML prion-infected Tga20 mice. First, HPMC in film form may act as a sustained releasing material for cpd-B. Previous study suggests that continuous administration is necessary for cpd-B to exert efficient effects due to the rapid clearance from the brain. Therefore, it is likely that the HPMC film extends the release of cpd-B and maintains the cpd-B concentration relatively longer even in a single subcutaneous administration. In fact, the water-soluble dye-releasing experiment showed that the HPMC film delayed the cpd-B diffusion rate by a half to the control (Fig. 3).

The other possibility is that HPMC may act on cpd-B-resistant prion variants, which were observed in the brain of RML prion-infected mice treated with cpd-B alone in the previous study [10]. These prion variants showed the di-glycosylated prion protein predominant type similar to 263K prion which is sensitive to HPMC treatment. Oelschlegel and Weissmann have reported that the RML prion is segregated into several sub-strains under the selective pressure of swainsonine [36]. Since the disease progression associated with the emergence of drug-resistant prion variants is an obstacle to treatment, the combination of cpd-B and HPMC may be a good example of overcoming this obstacle.

In contrast to the RML prion-infected mice, no synergistic effects by the combination of cpd-B and HPMC were achieved in 263K-infected Tg7 mice. In the previous literature, the emergence of distinct prion variants from 263K prion has not been reported. Since the drug resistance and adaptation of prion strains have been mainly studied in RML strains, whether the 263K prion variants emerge remains enigmatic. Considering no combination effects in 263K prion-infected Tg7 mice, it may be presumable that the synergistic effect in RML prion-infected Tga20 mice is related to the controlled cpd-B release rather than the suppression of prion variants. To confirm this, we need to verify the pharmacokinetics of cpd-B in Tga20 mice treated with HPMC film containing cpd-B in the future.

To address the diversity of prion strains, including human prions and their interactions [37, 38], multiple modalities comprising easier administration and sustained effect are required. Additionally, we also observed the anti-prion effect of HPMC depends not only on the prion strain but also on the mouse strain [14]. Recent structural analyses of a wide variety of PrPres including the RML and 263K strain [39] by cryo-electron microscopy have detected dissimilarities in their structural arrangements. Consequently, compounds designed considering those variations in prion strains will be developed broadening the modalities.

In conclusion, synergistic anti-prion effect in RML prion-infected Tga20 mice was observed by the combination treatment with two anti-prion compounds complementary in efficacy against prion strains, chemical properties, pharmacokinetics, and physical properties.

### Supplementary Information

Below is the link to the electronic supplementary material.

Supplementary file1 (DOCX 235 KB)

## Data Availability

All relevant data are within the paper and its supporting information files.

## Abbreviations

cpd-B: 1-(4-(Oxazole-5-yl)phenyl)-2-((pyridine-4-yl)methylene)hydrazine
HPMC: Hydroxypropyl methylcellulose
HR: Hazard ratio
N: Number of mice
SD: Standard deviation
Tg: Transgenic
