# Supplementary material for: Combination of Styrylbenzoazole Compound and Hydroxypropyl Methylcellulose Enhances Therapeutic Effect in Prion-Infected Mice
Source: Mol Neurobiol. 2023 Dec 20;61(7):4705–11. doi: 10.1007/s12035-023-03852-4 (PMC11236910; doi:10.1007/s12035-023-03852-4)
Supplement: Supplementary file 1 — Supplementary file1 (DOCX 235 KB) [file 12035_2023_3852_MOESM1_ESM.docx]

**Supporting**

**Combination of styrylbenzoazole compound and hydroxypropyl methylcellulose enhances therapeutic effect in prion-infected mice**

Kenta Teruya^1*^, Ayumi Oguma^1^, Sara Iwabuchi^1^, Keiko Nishizawa^1^, Katsumi Doh-ura^1,2^

^1^ *Graduate School of Medicine, Tohoku University, Sendai 980-8575, Japan*

*^2^Faculty of Medical Science & Welfare, Tohoku Bunka Gakuen University, Sendai, Miyagi,* Japan

* Corresponding author. Kenta Teruya

Department of Neurochemistry, Graduate School of Medicine, Tohoku University

2-1 Seiryo-machi, Aoba-ku, Sendai 980-8575, Japan

Tel: +81-22-717-8232, Fax: +81-22-717-7656 E-mail: kenta.teruya.d4@tohoku.ac.jp


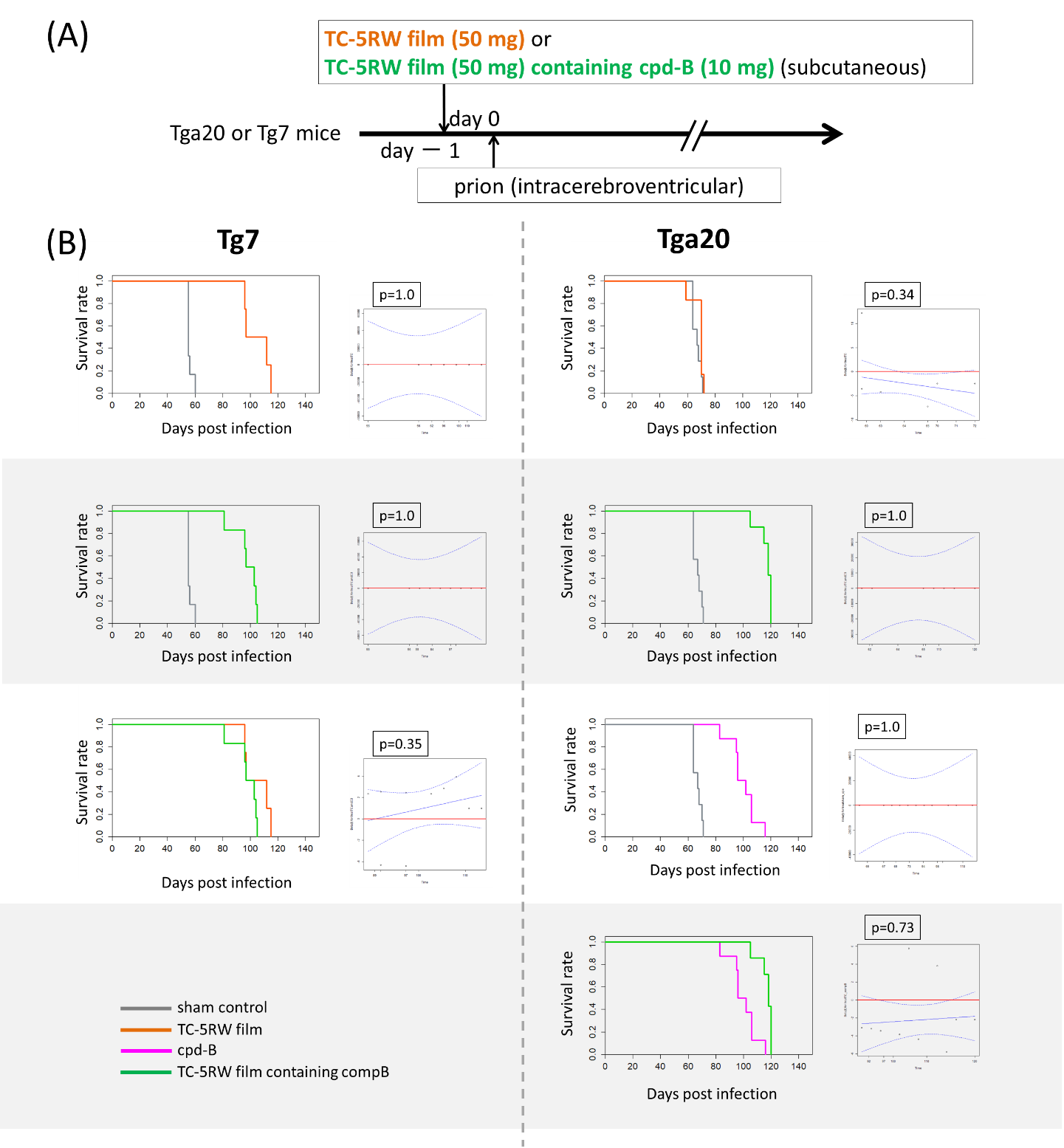


**Fig. S1** Evaluation of proportional hazards assumption for TC-5RW film and cpd-B doped film to prion infected mice

1. Timelines for evaluation TC-5RW film and cpdB-loaded film to prion infected mice.
2. Kaplan-Meier graphs of the TC-5RW films subcutaneous administration to prion-infected mice are shown, in conjunction with plots of the scaled Schoenfeld residuals (y-axis) against incubation time (days; x-axis) for the Cox’s proportional hazards model fitted to each dataset. Each Schoenfeld residual plot has a smoothing spline curve (df = 2; solid curve) overlaid with a 95% confidence interval (dashed curves). Note: The proportional hazards assumption is not violated, because there are no obvious trends in the residual plots for the treatment groups (i.e., the residuals are independent of time). This is also supported by the corresponding Grambsch-Therneau tests (p > 0.05). Data were analyzed in R using the ‘cox.zph’ function from the R package ‘survival’.
